# Does Cachexia Matter for Glioblastoma Multiforme?

**DOI:** 10.3390/cancers18020333

**Published:** 2026-01-21

**Authors:** Ryan Kelly, Lydia Henderson, Ishan Roy

**Affiliations:** 1 Shirley Ryan AbilityLab, Chicago, IL 60611, USA; 2 Department of Physical Medicine and Rehabilitation, Feinberg School of Medicine, Northwestern University, Chicago, IL 60611, USA; 3 Robert H. Lurie Comprehensive Cancer Center, Northwestern University, Chicago, IL 60611, USA

**Keywords:** cachexia, rehabilitation, brain cancer

## Abstract

Cachexia is a muscle-wasting syndrome that has a 50% overall prevalence across all cancers and is known to affect both survival and quality of life. However, its measurement, classification, and impact in individuals with primary brain tumors is unclear. Now, evidence is emerging that cachexia has a direct effect on both clinical and physical function outcomes for individuals with glioblastoma multiforme (GBM). Herein, we outline a standardized approach to the diagnosis of cachexia in the GBM population, incorporating several available clinical tools to ensure the link between clinical prognosis and quality of life.

## Simple Summary

People with cancer often experience muscle loss, weakness, and fatigue. This condition, known as cachexia, has been poorly recognized and measured in patients with brain tumors. Herein, we explain why cachexia matters for people with glioblastoma and how it can affect daily function, quality of life, ability to tolerate cancer treatments, and survival. However, simply tracking body weight may not be enough, as muscle loss and functional decline can occur even when weight appears stable. We describe practical ways clinicians can better identify cachexia using a combination of blood tests, imaging, physical function assessments, and patient-reported symptoms. Importantly, early recognition of cachexia can open the door to supportive treatments such as nutrition counseling, exercise-based rehabilitation, symptom management, and palliative care. Addressing cachexia alongside tumor treatment may help patients remain more independent, tolerate therapy longer, and maintain a better quality of life.

## 1. Introduction

Cachexia is a muscle-wasting syndrome that has a 50% overall prevalence across all cancers and is known to affect both survival and quality of life [1]. It is a muscle-wasting syndrome driven by a complex inflammatory process that results in coordinated dysfunction of multiple organ systems, including the heart, liver, brain, and pancreas, and commonly affects patients with cancer and other chronic conditions. Cachexia leads to progressive functional decline, weakens the efficacy of chemotherapy, and accounts for 20% of all cancer deaths [2]. Clinically, diagnosing cachexia can prove difficult when considering the existence of multiple criteria and screening measures, and the overlap between the similar diagnoses of frailty, malnutrition, and perhaps most frequently, sarcopenia (see Table 1 for a summary of diagnostic tools) [3]. Sarcopenia refers to the loss of skeletal muscle quantity or quality, loss of strength, and decreased functional performance [4]. Although the two syndromes often overlap, cachexia is defined by inflammatory and metabolic drivers linked to chronic disease that are not necessarily present in sarcopenia. In sarcopenia, muscle atrophy develops gradually over years, often stabilized or reversed with progressive resistance training and adequate protein intake [5]. Cachexia progresses rapidly, frequently over weeks or months, and is characterized by simultaneous muscle and fat loss accompanied by systemic inflammation, anorexia, and fatigue. One of the most common diagnostic approaches for cachexia measures unintentional weight loss using the Fearon Criteria, defined as, “…weight loss greater than 5%, or weight loss greater than 2% in individuals already showing depletion according to current bodyweight and height (body-mass index [BMI] < 20 kg/m^2^) or skeletal muscle mass (sarcopenia)” [6]. Additionally, there is increasing evidence for the inclusion of functional measures, inflammatory markers, biochemical markers of nutrition, and nutritional impact symptoms (NIS) (as outlined in Table 1) when considering a diagnosis of cachexia [7,8].

## 2. Epidemiology and Challenges of Identifying Cachexia in GBM

The prevalence of cachexia in patients with glioblastoma multiforme (GBM) is difficult to approximate and somewhat controversial. It has been estimated to range from 11 to 71% [20]. In one study, the presence of overweight and obese classifications in GBM patients was noted at the time of diagnosis, while significant weight loss was noted only after treatment initiation [21]. However, the measurement of weight loss alone may be a poor characterization of cachexia, failing to address muscle bulk and functional status in this population. In fact, the Fearon Criteria has limited efficacy in predicting survival in patients with GBM, while more complex longitudinal phenotyping approaches identify cachexia and have an impact on clinical and functional outcomes in patients with GBM [22].

In the absence of a standardized approach to detecting cachexia in GBM, clinicians may rely on muscle loss measurements that can be misappropriated as cachexia or sarcopenia. For example, while not diagnostic of cachexia, many studies have used temporal muscle thickness (TMT) to evaluate for sarcopenia in the brain injury population, including those with GBM, perhaps because it is easily measured using head CT or MRI scans routinely obtained to monitor disease progression. TMT has been shown to correlate with skeletal muscle area, supporting its use in the evaluation of muscle mass [23]. Potentially due to its relative ease of measurement in static imaging studies, low muscle mass is more often studied than cachexia, which requires measurement over time. However, the term sarcopenia has been misappropriated in prior studies of the GBM population to mean low muscle mass, while the consensus definition for sarcopenia also includes thresholds for decline in muscle strength and physical function [24]. Thus, while TMT can be a helpful static screening tool to measure muscle mass, it alone is not an appropriate diagnostic tool for cachexia or sarcopenia. Nevertheless, low muscle mass in patients with GBM has been linked to poor tolerance of oncologic treatment, with those affected demonstrating a higher likelihood of early discontinuation of chemotherapy and radiation, and a greater incidence of being prescribed steroids, and they are more often transferred to inpatient palliative care [25]. Conversely, a higher TMT is correlated with higher completion rates of the Stupp protocol, a standard treatment regimen for GBM [26]. Another study found that progression-free survival was shorter in patients with cancer who fell into the lowest sex-specific quartiles of muscle mass (8.4 months compared to 5.1 months) [25]. Low BMI patients with GBM have demonstrated shorter overall survival, and those with low TMT have significantly worsened overall survival as well as progression-free survival [26,27,28,29]. There is at least one study that combines both low muscle mass and decreased functionality in predicting survival outcomes in patients with GBM, demonstrating a median overall survival of 13.9 months compared to 5.8 months for patients in the high-risk group based on a modified frailty score [28]. Furthermore, the presence of low muscle mass has functional implications for patients with GBM, as it has been linked to a decline in both quality of life and overall function as measured using the Karnofsky Performance Status scale [26,28].

## 3. Treatment and Tumor Related Confounders

Despite the association between proxy measures of muscle mass and poor prognosis in patients with GBM, the treatment of cachexia in this population is rarely discussed for a multitude of potential reasons. GBM is rapidly progressive, with a five-year survival rate below 10%; thus, aggressive surgical management, immunotherapy, and adjuvant treatments are often directed towards the primary disease [30]. Clinical evaluation of these patients may be tailored towards primary tumor symptoms, many of which can overlap with cachexia: fatigue, decreased appetite, nausea, weakness, etc. Additionally, standard treatments for GBM are often associated with symptoms that are difficult to neatly categorize as treatment adverse effects or systemic effects of cachexia. For example, temozolomide is commonly associated with nausea, vomiting, gastrointestinal upset, and fatigue [31]. Bevacizumab may cause weight fluctuations, swelling, and nausea [32,33]. Post-surgical and radiation fatigue, cerebral edema, nausea, and cognitive changes are common in the GBM population, and adjuvant treatments may cloud the precise etiology of neurocognitive symptoms and NIS [34].

Corticosteroids, particularly dexamethasone, may further complicate the identification of cachexia in patients with GBM. NIS may come from brain injury from the presence of a tumor, brain injury from treatment effects (post-surgical or radiation), and/or systemic inflammation that directly affects the hypopituitary axis [35,36]. Dexamethasone, a standard of care in GBM-related cerebral edema, may mask NIS by transiently stimulating appetite and result in an increase in body weight. However, several studies have shown that steroids do not reverse the inflammatory drivers of cachexia and are associated with an increase in body fat mass, not skeletal muscle, and may potentially lead to muscle atrophy and weakness, further exacerbating functional decline despite apparent weight stability or gain [37,38,39].

## 4. A Practical Assessment of Cachexia in GBM

Given the complexities of differentiating between disease-related symptoms, treatment-related adverse effects, and cachexia, more objective assessments of GBM cachexia that involve the analysis of inflammatory markers are likely needed. Already, studies suggest that neutrophil-to-leukocyte ratio, prognostic nutritional index, and c-reactive protein are each useful tools in cachexia evaluation in patients with glioma [12]. However, unlike change in weight over time, inflammatory markers are static biochemical snapshots with limited application in isolation, revealing little about longitudinal progression or functional status in patients with cachexia [11,22,40,41,42]. Similarly, the image-based muscle assessments discussed above are limited in assessing cachexia in isolation. More sophisticated machine learning models, such as dynamic time warping (mentioned above), account for longitudinal cachexia progression and have more recently demonstrated improved prognostic capacity for patients with GBM cachexia [22]. Thus, the recognition of cachexia patterns, rather than any single measure, is useful in moving clinicians to action to avoid death and disability from GBM cachexia.

With respect to a functional decline from cachexia, a comprehensive history remains key in identifying areas of impairment. Preserved function, particularly as it relates to independence with activities of daily living (ADLs), has been linked to improved quality of life and treatment adherence in cancer patients [43,44,45]. There are numerous patient reported outcomes (PROs), including the functional assessment of anorexia-cachexia therapy (FAACT) scale and functional assessment of cancer therapy general (FACT G) scale which assess quality of life, while the European Organization for the Treatment and Research of Cancer Quality of Life Questionnaire (EORTC QLQ-C30) offers more specifics on functional independence [14,15,16]. Clinical assessments of function, including the 30 s sit-to-stand and the timed up and go tests, may also provide insight into functionality [46].

In clinical practice, a comprehensive evaluation for a patient with GBM with concern for cachexia should begin by considering the patient’s diagnosis, treatment history, PROs, available serum markers, and physical measurements. Then, a comprehensive history, with particular attention to functional independence in ADLs followed by a physical exam to assess functional strength and mobility, should be conducted. A detailed summary of some relevant assessments is provided in Table 1. More than any one metric, the synthesis of the above data points can better inform clinical decision making in constructing a comprehensive plan, improve longitudinal tracking and machine learning models, and guide the selection of appropriate therapeutic interventions (Table 2).

## 5. Conclusions

Early recognition of the clinical patterns of cachexia, comprehensive clinical evaluation, and interdisciplinary collaboration are necessary to promote greater treatment outcomes while preserving the function and quality of life in GBM patients with cachexia.

Building on this need for earlier recognition and coordinated care, future directions in cancer cachexia may focus on early detection and longitudinal assessment, precision phenotyping, integration of machine learning approaches, and expansion of multimodal care models across oncology and rehabilitation disciplines. Advances in body composition assessment, such as computed tomography (CT), magnetic resonance imaging (MRI), dual-energy X-ray absorptiometry (DXA), bioimpedance, and bedside ultrasound, enable objective quantification of skeletal muscle and fat loss. These imaging-based metrics may be paired with clinical and biochemical data to create predictive models that use machine learning to identify high-risk patients before overt wasting develops, thereby supporting proactive intervention and the early targeting of metabolic and functional decline.

In parallel, broader biomarker panels, including endocrine markers such as thyroid hormones and testosterone, may further enhance precision monitoring and guide individualized therapy [49,50]. Among emerging biochemical targets, growth differentiation factor-15 (GDF-15) has drawn particular attention for its role in appetite suppression and energy balance, with early studies demonstrating improvements in weight, appetite, and activity levels in patients with cancer-associated cachexia [51].

Alongside these diagnostic and biochemical advances, tailored rehabilitation and exercise-based programs, not yet widely integrated into standard oncologic care, should be considered to help preserve muscle bulk and prevent functional decline in patients with cachexia. Emerging digital health tools, such as wearable devices capable of tracking activity, muscle function, and weight trends, may soon enable real-time surveillance, with artificial-intelligence systems flagging early functional decline and prompting timely intervention.

Cachexia, despite its relatively poor characterization within the GBM population, exists as a major threat to patient quality of life, treatment adherence, and survival outcomes. There remains a large need for earlier detection and integrated, multimodal strategies that combine emerging modalities to improve patient-centered outcomes.

## Figures and Tables

**Table 1:** Tools for the diagnosis of cachexia in patients with GBM.

Domain	Assessment/Tool	Description	Benefits	Limitations
Clinical	Weight history, body mass index	Unintentional weight loss over time	Simple, widely used	May miss sarcopenic obesity or edema masking weight loss [9]
	Fearon criteria [6]	Consensus-based clinical definition	Standardized criteria for diagnosis	Does not evaluate functional status
Biochemical	C-reactive protein [10]	Marker of systemic inflammation	Accessible and widely used	Nonspecific; elevated in many GBM patients
	Neutrophil-to-leukocyte ratio [11]	Inflammation-related biomarkers	Obtained on routine labs	Nonspecific
	Prognostic nutritional index [12]	Malnutrition assessment using albumin and lymphocyte count	Easy to obtain	Currently with limited evidence
Functional	Barthel index [13]	Monitoring of activities of daily living	Objective, easy for patients to report	Not specific to cancer or cachexia evaluation
	FAACT, FACT G, EORTC QLQ-C30 [14,15,16]	Questionnaires to assess quality of life and nutrition	Simple, non-invasive	Patient-reported rather than clinician-assessed data
	Karnofsky performance status [17]	Functional status scoring system for cancer patients	Clinically useful for tracking performance in patients with GBM [18]	Subjective, affected by tumor-related symptoms
Imaging	CT/MRI-based muscle mass assessment	Cross-sectional cervical muscle area, temporal muscle thickness	Objective and quantitative	Requires advanced imaging
Metabolic	Bioelectrical impedance analysis [19]	Estimates body composition	Quick, non-invasive	Not available in all clinical settings

**Table 2:** Cachexia treatment options.

Treatment Type	Intervention	Description
Nutritional Support	Nutritional counseling	Personalized, can help optimize macronutrient and caloric needs
	Enteral or parenteral nutrition	Alternative forms of nutrition when patients have difficulty with oral intake
Pharmacologic	Appetite stimulants	Some options include olanzapine, dronabinol and cannabis derivatives, and mirtazapine
Physical Activity	Regular exercise	Aerobic exercise reduces systemic inflammation [47]. Resistance training preserves or rebuilds muscle mass and improves performance [48].
	Physical, occupational, and speech therapy	Improve functional status, mobility, and cognition/communication
Psychosocial	Psychology support	Address depression, anxiety, and coping
	Palliative care involvement	Align goals of care and decision making, symptom management, and support mechanisms for patients and caregivers

## Data Availability

No data was shared.
